# The Yeast P5 Type ATPase, Spf1, Regulates Manganese Transport into the Endoplasmic Reticulum

**DOI:** 10.1371/journal.pone.0085519

**Published:** 2013-12-31

**Authors:** Yifat Cohen, Márton Megyeri, Oscar C. W. Chen, Giuseppe Condomitti, Isabelle Riezman, Ursula Loizides-Mangold, Alaa Abdul-Sada, Nitzan Rimon, Howard Riezman, Frances M. Platt, Anthony H. Futerman, Maya Schuldiner

**Affiliations:** 1 Department of Molecular Genetics, Weizmann Institute of Science, Rehovot, Israel; 2 Department of Biological Chemistry, Weizmann Institute of Science, Rehovot, Israel; 3 Department of Pharmacology, University of Oxford, Oxford, United Kingdom; 4 Department of Biochemistry, University of Geneva, Geneva, Switzerland; 5 School of Life Sciences, University of Sussex, Brighton, United Kingdom; 6 National Centre of Competence in Research (NCCR) Chemical Biology, University of Geneva, Geneva, Switzerland; 7 The Joseph Meyerhoff Professor of Biochemistry at the Weizmann Institute of Science, Weizmann Institute of Science, Rehovot, Israel; Université de Montréal, Canada

## Abstract

The endoplasmic reticulum (ER) is a large, multifunctional and essential organelle. Despite intense research, the function of more than a third of ER proteins remains unknown even in the well-studied model organism *Saccharomyces cerevisiae*. One such protein is Spf1, which is a highly conserved, ER localized, putative P-type ATPase. Deletion of *SPF1* causes a wide variety of phenotypes including severe ER stress suggesting that this protein is essential for the normal function of the ER. The closest homologue of Spf1 is the vacuolar P-type ATPase Ypk9 that influences Mn^2+^ homeostasis. However in *vitro* reconstitution assays with Spf1 have not yielded insight into its transport specificity. Here we took an *in vivo* approach to detect the direct and indirect effects of deleting *SPF1*. We found a specific reduction in the luminal concentration of Mn^2+^ in *∆spf1* cells and an increase following it’s overexpression. In agreement with the observed loss of luminal Mn^2+^ we could observe concurrent reduction in many Mn^2+^-related process in the ER lumen. Conversely, cytosolic Mn^2+^-dependent processes were increased. Together, these data support a role for Spf1p in Mn^2+^ transport in the cell. We also demonstrate that the human sequence homologue, ATP13A1, is a functionally conserved orthologue. Since ATP13A1 is highly expressed in developing neuronal tissues and in the brain, this should help in the study of Mn^2+^-dependent neurological disorders.

## Introduction

Eukaryotic cells contain organelles that are essential for creating a diverse range of chemical microenvironments. One such organelle is the endoplasmic reticulum (ER), which serves as the entry site into the secretory pathway allowing folding and maturation of all secreted and membrane-bound proteins [1–4]. In addition, the ER is the main site of cellular lipid biosynthesis [5–8] and is important in regulating homeostasis of different ions [9,10]. Over the years, the budding yeast *Saccharomyces cerevisiae* was established as an excellent model for studying various aspects of ER function, but the full range of proteins that contribute to its normal function is still unknown [11]. Moreover, the function of many ER resident proteins, essential for maintaining organelle homeostasis, has not yet been characterized [12].

One such protein termed Spf1 (Sensitivity to *Pichia Farinosa* killer toxin 1), spans the ER membrane through its 12 transmembrane domains [13]. The absence of *SPF1* causes one of the most severe stress phenotypes in the ER, hinting at the central role of this protein in normal ER function [12]. In addition, *SPF1* expression is elevated following ER stress [14,15]. More generally, over the last decade many genetic screens on various ER functions have identified Spf1 as an important player. Indeed, loss of Spf1 seems to affect varied processes such as kinetics of degradation of the ER resident protein HMG2 [16], sensitivity to killer virus toxin [13], membrane composition [17], and more [12,18–27] (for the full list see Table S1). 

However, how one protein can affect such a myriad of processes remained elusive and a unifying theory was required to explain the drastic outcomes of its loss on ER function. Therefore, we sought to uncover the biochemical activity of Spf1. According to its sequence, Spf1 belongs to the family of P-type ATPases - a specific subgroup of the transport ATPase family. All transport ATPases are membrane-bound proteins that hydrolyze ATP to allow the transport of at least one substance across a biological membrane. Transport ATPases exist in all kingdoms: bacteria, fungi, plant and animals[28,29]. The subfamily of P-type ATPases are all multi-span transmembrane proteins that establish and maintain steep electrochemical gradients of key cations or phospholipids across membranes and are therefore vital to all eukaryotes and most prokaryotes. 

P-type ATPases are divided into 5 subgroups (termed P1-P5) based on sequence similarity [30]. The subgroups of P1-P3 ATPases include proteins that are responsible for the transport of a broad variety of cations. Type P4 ATPases form a distinct group based on sequence divergence and their proposed role is in transport/flipping phospholipid molecules rather than cations [31,32]. The most poorly characterized subgroup of ATPases is that of the P5s, which are expressed in eukaryotes from fungi to vertebrates and have a conserved core sequence that differs from all other subgroups. Spf1 is one of the two *Sacchromyces cerevisiae* P5 ATPases, along with Ypk9. While Spf1 localizes to the ER, Ypk9 is vacuolar [33–35]. Although Spf1 has been studied for more than a decade and extensive efforts have been made to identify its substrate, its molecular function remains unknown. 

Recently, an effort has been made to identify Spf1's substrate in reconstituted liposomes with no success [36]. Although *in vitro* reconstitution is often an essential step in defining the direct substrate of a transporter, this method also has its pitfalls and may be problematic for a variety of reasons. First, for a very large protein with 12 transmembrane domains, such as Spf1, it is very difficult to ensure that correct conformation is acquired within the membrane bilayer. Second, the synthetic lipid bilayer may not have the same biophysical characteristics as the membrane in which Spf1 normally functions. Third, a buffer most likely cannot identically recapitulate the ionic conditions in the cytosol or within the ER lumen. To this end, the difficulty to demonstrate a direct substrate for Spf1 using these methods suggests that we must examine its activity in its natural environment, in the cell, within the ER membrane. 

Recently, it was shown that the other P5 ATPAse in yeast, Ypk9, plays a role in Mn^2+^ tolerance [37]. Mn^2+^ is a biologically-important metal that is both a nutrient and a toxic element. Cells must therefore carefully control the uptake and intracellular trafficking of this ion. For this purpose, Mn^2+^ transporters must be present in each and every compartment of the eukaryotic cell. While transporters are known for the plasma membrane, intracellular vesicles, mitochondria, vacuole and the Golgi [38–40], the ER Mn^2+^ transporter remains elusive. Due to the absence of an ER transporter and the homology of Spf1 to Ypk9, we hypothesized that Spf1 might be the Mn^2+^ transporter of the ER. If so, the lumenal concentration of Mn^2+^ should be reduced in its deletion strains and elevated upon its overpexpression and changes in Mn^2+^ distribution in the cell should be at the basis for the entire spectrum of defects found in the absence of Spf1 function.

Here, we show that indeed Mn^2+^ levels are reduced in ∆*spf1* microsomes (ER-derived vesicles) and are increased in Spf1-overexpressing (OE) microsomes compared to WT. We also demonstrate that the activity of many Mn^2+^ requiring enzymes is affected in ∆*spf1* cells. Our results can not prove that Spf1 is the direct transporter of Mn^2+^ but demonstrate that Spf1 influences Mn^2+^ homeostasis in the cell. In addition we demonstrate functional conservation to its mammalian homologue ATP13A1, and provide the first evidence of the influence of ATP13A1 on Mn^2+^-dependent processes in mammalian cells.

## Materials and Methods

### Yeast Media and Growth Conditions

Yeast cells were grown at 30°C in either rich medium, YPD (1% Bacto-yeast extract (BD), 2% Bacto-peptone (BD) and 2% dextrose (Amresco)) or synthetic medium, SD (0.67% yeast nitrogen base with ammonium sulfate and without amino acids (CondaPronadisa) and 2% dextrose (Amresco), containing the appropriate supplements for plasmid selection) [41]. When needed for selection, the media was supplemented with G418 (200 ug/ml, Calbiochem) or nourseothricin (Nat) (200 ug/ml WERNER BioAgents). In cases where G418 was required in an SD-based medium, yeast nitrogen base without ammonium sulfate (conda Pronadisa) was added and supplemented with mono-sodium glutamate (Sigma). When stated, drugs were added to YPD media in the following concentrations: 0.05 ug/ml Terbinafine (ENZO BIOMOL), 100 ug/ml Aureobasidin A (Clontech).

Except from the filipin staining, all experiments perform at logarithmic stage, for that purpose yeast cells were grown overnight in the appropriate media and then back-diluted before the specific assay. In case of the filipin staining, strains were grown overnight to stationary phase and then samples were stained for sterol, using the filipin dye (Sigma). 

### Yeast Strain and Strain Construction

All strains were constructed on the S288C (BY4741) genetic background [42]. We used deletion strains from the deletion library collection harboring a G418 resistance cassette [43]. To ensure the validity of a deletion, primers were used to check for lack of a remaining copy of the gene. The construction of GFP tagged proteins was performed by PCR-mediated homologous recombination using pYM-N21-natNT2-TEF-yeGFP marker cassettes for N’ tagging [44]. All transformations (of plasmids [27,45] or for the purpose of genomic integration) were performed using a standard PEG/LiAC protocol as previously described [46]. Strains, plasmids and primers used in this study are listed in Tables S2, S3 and S4.

### Microsome Preparation

Subcellular fractionation was performed as described [47]: Strains grown to logarithmic stage in 1liter of YPD medium were harvested and washed once in 100 mM Tris-HCl pH 9.4, 10 mM DTT and converted to spheroplasts by incubation with 3.4 mg Zymolase (MP Biomedicals). Spheroplasts were isolated by centrifugation through a 0.8 M sucrose cushion and were frozen in −80°C. Pellets were re-suspended in lysis Buffer (0.1 M sorbitol, 20 mM HEPES pH 7.4, 50 mM KAc, 2 mM EDTA, 1 mM DTT, 1 mM PMSF) and were subjected to 25 strokes in a Dounce homogenizer and the resulting supernatant was ultracentrifuged at 27,000 g (Ti60 rotor; Beckman Instruments, Palo Alto, CA) for 10 min. at 4°C. Pellet was re-suspended in lysis buffer and was placed on a sucrose gradient consisting of 2 steps of 1.2 M and 1.5 M sucrose in lysis buffer. The gradient was centrifuged at 100,000 g (SW41 rotor; Beckman Instruments) for 1 hr at 4°C. Membranes were collected from the gradient interface and were washed twice in reaction buffer (20 mM HEPES pH 6.8, 150 mM KAc, 5 mM MgAc, 250 mM Sorbitol) followed by re-suspension in same buffer. Total microsome protein concentration was determined using BCA reagent (Thermo Scientific).

### ICP-MS for metal ions measurements

Microsomes samples were treated as previously described [48]. In brief, samples were incubated with 3% nitric acid for overnight reaction at 98°C, following with metal ions content measurements using Inductively Coupled Plasma Mass Spectrometer (ICP-MS). Samples were measured metal ions isotopes (Fe-56, Cu-63, Zn-66 and Mn-55) by Agilent 7500 Series ICP-MS (Department of Chemistry, University of Sussex), and Ge-72 was used as internal standard. Calibration solutions for Fe were prepared between 0 and 1.0 µg/ml; calibration solutions for the other elements were prepared between 0 and 0.1 µg/ml. Metal ions content were normalized with protein concentration. Measurements included three biological repeats for WT microsomes, four repeats for ∆*spf1* microsomes and one for OE-Spf1p microsomes.

### Flow cytometry

Cells were grown over night in SD complete and were back diluted to logarithmic phase. Level of GFP fluorescence was detected in living cells by BD™ LSR II Flow Cytometer System and the analysis was performed using appropriate settings with FCS Express De Novo Software. 50000 cells were detected in each sample and the same gate according to cell size was included in the analysis for all samples.

### Fluorescence microscopy

Images of live cells were acquired at room temperature using an Olympus IX71 microscope controlled by Delta Vision SoftWoRx 3.5.1 software with either X20 air (for mammalian cells) or X100 oil (for yeast cells) lenses (unless stated otherwise). Images were captured by a Phoetometrics Coolsnap HQ camera with excitation at 490/20 nm and emission at 528/38 nm (GFP), excitation at 555/28 nm and emission at 617/73 nm (RFP), excitation at 492/18 nm and emission at 535/30 nm (YFP) or excitation at 360/40 nm and emission at 457/50 nm (DAPI). Representative images from several independent experiments are shown as the results.

Fillipin (Sigma) staining of yeast cells and Hoechst (Invitrogen) and Cholera toxin (Molecular PROBES) staining of mammalian cells performed according to the manufacturers.

### Western blotting of total cellular protein extracts

#### Yeast cells

Yeast protein extraction were performed as previously described [49]. In brief, 1.5 O.D.600 of mid-logarithmic yeast cells were harvested, and re-suspended in 0.1M NaOH and incubate on ice for 10 min. Cells were then centrifuged for 1 min. at 14,000 rpm, and the pellet was resuspended in 50 μl of loading buffer (0.05 M Tris-HCl, pH6.8, 10% glycerol, 2% SDS, 5% β-mercaptoethanol, 0.1% bromophenol blue). The samples were then incubated at 100°C for 5 min., and centrifuged for 1 min. at 14,000 rpm. 5 μl from the supernatant of the samples was resolved on 3-8% polyacrylamide gels (BioRad), transferred to nitrocellulose membranes blots (BioRad), and probed with rabbit polyclonal α-GFP (ab290, Abcam), rabbit polyclonal α-Gas1 (kindly provided by Randy Schekman), or rabbit polyclonal α-Yps1 (kindly provided by Yves Bourbonnais). Secondary antibodies consisted of goat α-rabbit conjugated to IRDye800 (LI-COR Biosciences), and were scanned for infrared signal using the Odyssey Imaging System (LI-COR Biosciences).

#### Mammalian cells

Cells were washed in PBS and then suspended and vortexed in cold lysis buffer (50 mM TrisHCl pH 8, 150 mM NaCl, 1% NP-40, 0.5 mM EDTA) with protease inhibitors (x2 Complete protein inhibitor cocktail (Roche)), and kept on ice for 30 minutes and vortexed every 5 min. Lysates were centrifuged for 15 min. at 14,000 rpm at 4°C. The same amount of protein, as measured by using BCA reagent (Thermo Scientific) was resolved on 4-20% polyacrylamide gels (NuSep), transferred to nitrocellulose membranes blots, and probed with rabbit polyclonal α-GAPDH (ab9485, Abcam) or with rabbit polyclonal α-GRP78 BiP (ab21685, Abcam). Secondary antibodies consisted of goat α-rabbit conjugated to IRDye800 (LI-COR Biosciences), and were scanned for infrared signal using the Odyssey Imaging System (LI-COR Biosciences). 

### Mammalian cell culture and transient transfection

HeLa cells were grown in DMEM (Biological Industries) supplemented with 10% fetal bovine serum (Hyclone) and 1% L-Glutamine (GibcoBRL) and a mixture of antibiotics (100 u/ml penicillin and 0.1 mg/ml streptomycin). For transient transfections of siNon target (siGENOME SMARTpool, Dharmacon) and siATP13A1 (siGENOME SMARTpool, Dharmacon), cells were plated for 30-50% confluent. Transfections were performed using lipofectamin (Invitrogen) and according to the manufacturer. Cells were then analyzed 72h following the tranfection. 

### RNA extraction

Cells were transfected with siRNA and after 72 hours, RNA purification was performed using the TriReagent (MRC) according the manufacturer. cDNA was generated using the SuperScript® III First Strand Synthesis kit (Invitrogen). mRNA level measurements were performed using quantitative real-time PCRs (qPCR) that were performed in a StepOnePlus™ Real-Time PCR system (Applied Biosystems) using Fast SYBR® Green Master Mix, with RPLPO gene as reference. Relative expression results (RQ) were calculated using the StepOne software.

### Lipidomics

#### Sphingolipid measurements in yeast

Sphingolipids were extracted from logarithmic phase cells (OD_600_ 1-2) and measured as described [50] using methylamine treatment with some modifications. Glucosylceramide was an internal standard as yeast complex sphingolipid standards are not available commercially. Therefore, the measurements represent relative amounts, which can be compared between strains, rather than absolute quantities. The total amounts of sphingolipids were normalized based on total lipids extracted as determined by measurement of inorganic phosphate by the procedure on the Avanti Polar Lipids website (http://www.avantilipids.com/index.php?option=com_content&view=article&id=1686&Itemid=405). Sphingolipids were measured using the described transitions [51] after direct infusion of extracts with internal standards using nanoflow (Advion Nanomate) on a TSQ Vantage Triple quadrupole (Thermo Scientific) and quantified using standard curves.

#### GlcCer measurements in mammalian cells

HeLa cells were transfected for 72 hours with siRNA. Cells were washed once with cold sterile PBS and collected using 1 ml of sterile water. The samples were then lyophilized for 48 hours. For GlcCer measurements lipid extracts were prepared using a modified MTBE method as previously described [52]. Briefly, lyophilized cells were resuspended in 100 μl H_2_O. The cell suspension was transferred into a 2 ml Eppendorf tube. 360 μl methanol, the internal standard (100 pmol Glucosyl C8:0 Cer, Avanti Polar Lipids) and 1.2 ml MTBE were added. Samples were vortexed for 10 min at 4 °C followed by an incubation for 1 h at room temperature on a shaker. Phase separation was induced by 200 μl MS-grade water. After 10 min incubation, samples were centrifuged at 1000 g for 10 min. The upper phase was transferred into a glass tube and the lower phase was reextracted with 400 μl artificial upper phase (MTBE / methanol / H_2_O 10:3:1.5). The total organic phase recovered from each samples was pooled, split into three parts and dried. One part was treated by alkaline hydrolysis to enrich for sphingolipids as described before [52] and the other two aliquots were used for glycerophospholipid/phosphorus assay respectively. Tandem mass spectrometry for the identification and quantification of GlcCer molecular species was performed by direct infusion using multiple reaction monitoring with a TSQ Vantage Triple Stage Quadrupole Mass Spectrometer (Thermo Scientific) equipped with a robotic nanoflow ion source (Nanomate, Advion). GlcCer concentrations were calculated relative to the internal standard and then normalized to the total phosphate content of the total lipid extract as described before [52].

### Immunostaining

HeLa cells were cultured on round glass slides for one day. Semi confluent cells were washed with PBS and fixed with 4% paraformaldehyde (PFA) for 20 min and perforated with 0.01% Triton-X solution for 5 min. Cells were blocked by 10% normal horse serum (NHS) (Vector Laboratories) for 30 min and developed with ATP13A1 antibody (Atlas Antibodies) diluted in PBS containing 1% NHS for overnight at 4°C and stained with CF568 goat anti-rabbit IgG (Biotium) and Hoechst 33342 (Molecular probes). Confocal microscopy was performed using an Olympus IX 81 Fluo-View 1000 microscope and a UPLSAPO 60x objective and images were processed and analyzed using FV-1000 software.

## Results

### Spf1 is required for Mn^2+^ homeostasis in the cell

**Figure 1:**
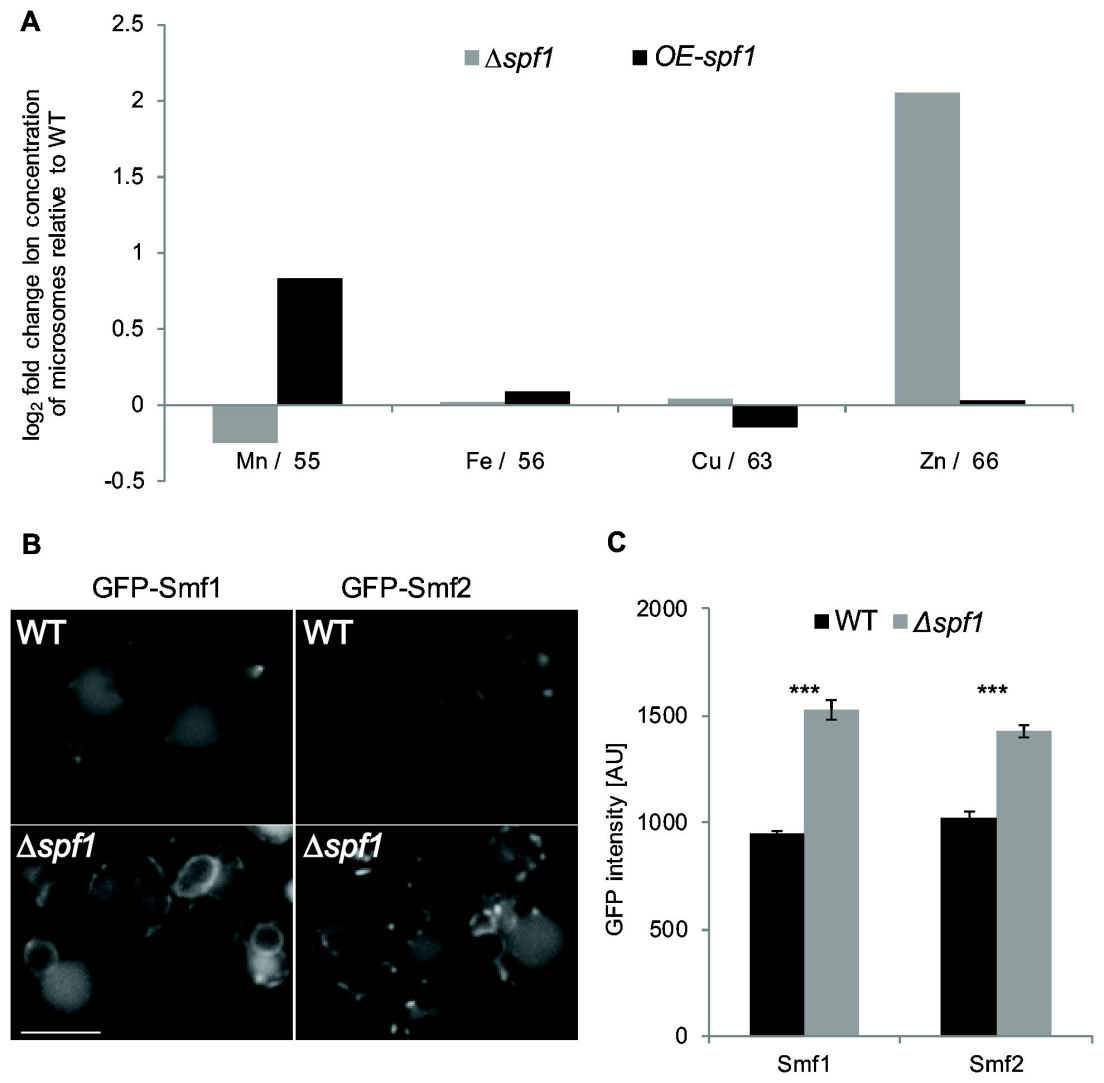
Spf1 affects the cellular distribution of Mn^2+^. (**A**) Metal content of microsomes from Δ*spf1*, OE-Spf1 and WT yeast was determined using ICP-mass spectrometry. By looking at the fold change of ion concentration of Δ*spf1* or OE-Spf1 microsomes relative to WT it can be seen that Mn^2+^ is the only ion that decreases in Δ*spf1* and increases in microsomes of OE-Spf1. (**B**) Representative fluorescent images of cells expressing GFP tagged Smf1 or Smf2 proteins which act in the uptake and intracellular trafficking of Mn^2+^. In Δ*spf1* these reporters of luminal Mn^2+^ are stabilized and can be seen on the cell surface or intracellular vesicles and less in the vacuole. Scale bar: 5µm. (**C**) Intensity measurements by flow cytometry of GFP tagged Smf1 or Smf2 proteins indicate higher levels of these proteins in *∆spf1* cells compared to WT. *** P value<0.001.

As a P-type ATPase, Spf1 most likely functions as an ion transporter [28], we therefore first measured the levels of a variety of bivalent ions in microsomes from strains deleted for Spf1 (∆*spf1*) or strains over expressing Spf1 (OE-Spf1) relative to WT strains. Mn^2+^ was the only ion that decreased in Δ*spf1* microsomes and increased concomitantly in OE-Spf1 microsomes (Figure 1A). 

We wanted to know whether these changes in Mn^2+^ levels are not only statistically significant but are indeed enough to have a physiological effect. To this end we examined the Mn^2+^ transporters Smf1 and Smf2 that are known to sense luminal Mn^2+^ [53]. In WT cells, under physiologic Mn^2+^ levels the majority of newly synthesized Smf1/2 proteins are directly targeted to the vacuole for degradation [40]. This constitutive degradation maintains these transporters at steady state levels that are just enough to supply the cell with essential Mn^2+^. Depletion of Mn^2+^ inside the ER lumen causes a change in Smf1/2 conformation rendering them incompetent to bind the degradation chaperone Bsd2 thus resulting in accumulation of these proteins on the cell surface and intracellular vesicles [38,54–56]. Hence, the level and localization of these two transporters can be used as probes to report on the levels of Mn^2+^ in the ER lumen. Indeed, *∆spf1* cells that express GFP tagged Smf1 and Smf2 proteins show much stronger localization to the cell surface and intracellular vesicles and less to the vacuole compared to WT (Figure 1B) and an increased abundance (Figure 1C). Taken together we concluded that Spf1 affects the concentration of the essential ion, Mn^2+^ in the ER lumen to an extent that is physiologically relevant.

It has been shown that a deletion of *SPF1* causes an enormous number and range of phenotypes. Indeed, intracellular changes in Mn^2+^ homeostasis should affect many processes in the cell due to the existence of different enzymes that are known to require Mn^2+^ for their normal function [40]. To this end, we decided to see whether the variety of phenotypes observed in the absence of *SPF1* can be accounted for by loss of activity of Mn^2+^-dependent enzymes in the ER lumen or an increase in activity of the cytosolic enzymes.

### The biosynthesis of lipids is influenced in ∆*spf1* cells

**Figure 2:**
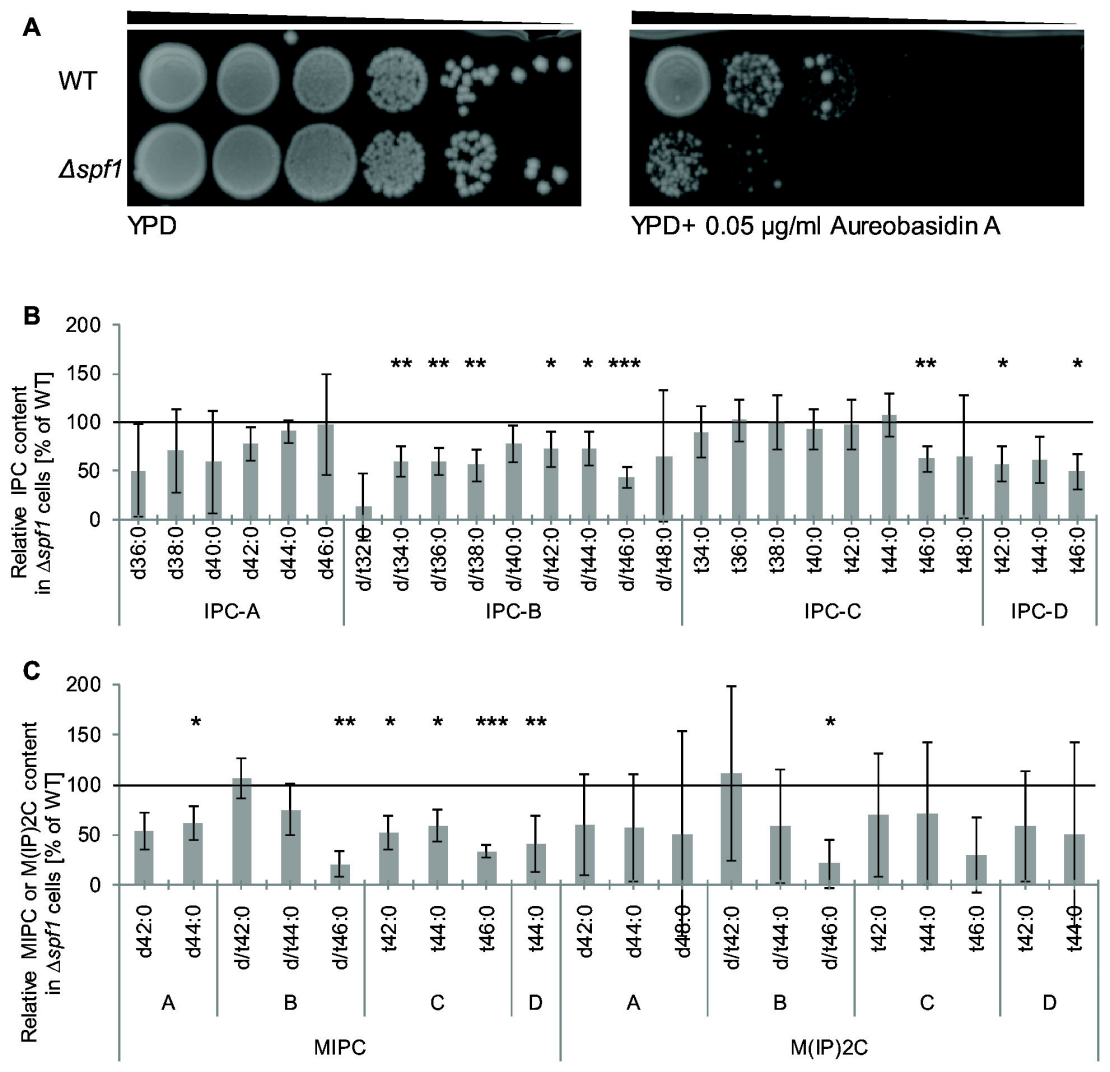
Biosynthesis of sphingolipids is interrupted due to deletion of SPF1. (**A**) WT and Δ*spf1* cells were plated in serial dilution on YPD plates without and with Aureobasidin A, an IPC synthase inhibitor. Loss of SPF1 increases the sensitivity to this inhibitor. (**B**) The levels of different types of IPC, were measured by mass spectrometry and are mostly lower in Δ*spf1* cells compared to WT. (**C**) The levels of different types of MIPC and M(IP)2C, were measured by mass spectrometry and are mostly lower in Δ*spf1* cells compared to WT. * P value<0.05, ** P value<0.01, *** P value<0.001.

Mn^2+^ is an essential cofactor for a large variety of enzymes, and in particular for enzymes that are involved in lipid biosynthesis and glycosyltransferases. One such enzyme is the essential inositol-phosphoceramide (IPC) synthase, Aur1, which is the key enzyme in the biosynthetic pathway of complex sphingolipids in yeast [57,58]. Aur1 has an active site facing the lumen of the endomembrane system [59] and therefore requires luminal Mn^2+^ for its activity. The subsequent enzymes, IPC:mannosyltransferase and MIPC:inositolphosphate transferase, are also likely to have active sites facing the luminal compartment and may require Mn^2+^ as well. Aureobasidin A (AbA) is a specific inhibitor of Aur1 activity and hence leads to cell death [60,61]. We found that Δ*spf1* cells were much more sensitive to AbA than WT cells (Figure 2A) suggesting that Aur1 activity is already reduced in these cells.

To investigate whether indeed Aur1 activity is reduced upon deletion of *SPF1* we measured the level of the three classes of complex sphingolipids in *∆spf1* and WT strains: inositol-phosphoceramide (IPC), mannose-inositol-phosphoceramide (MIPC), and mannose-(inositol phosphate)_2_-ceramide (M(IP)_2_C) [5,62]. After lipid extraction and mass spectrometry, we found that, in general, the levels of these three types of sphingolipids were reduced in *∆spf1* cells relative to WT controls. More specifically, most of the IPC types in *∆spf1* cells were decreased in a statistically significant manner (Figure 2B). The majority of the MIPC levels were decreased in a statistically significant manner (Figure 2C) in *∆spf1* cells, while M(IP)_2_C levels also tend to be lower upon this deletion although more variable (Figure 2C). These results strongly suggest that Aur1 is less functional in these cells. 

**Figure 3:**
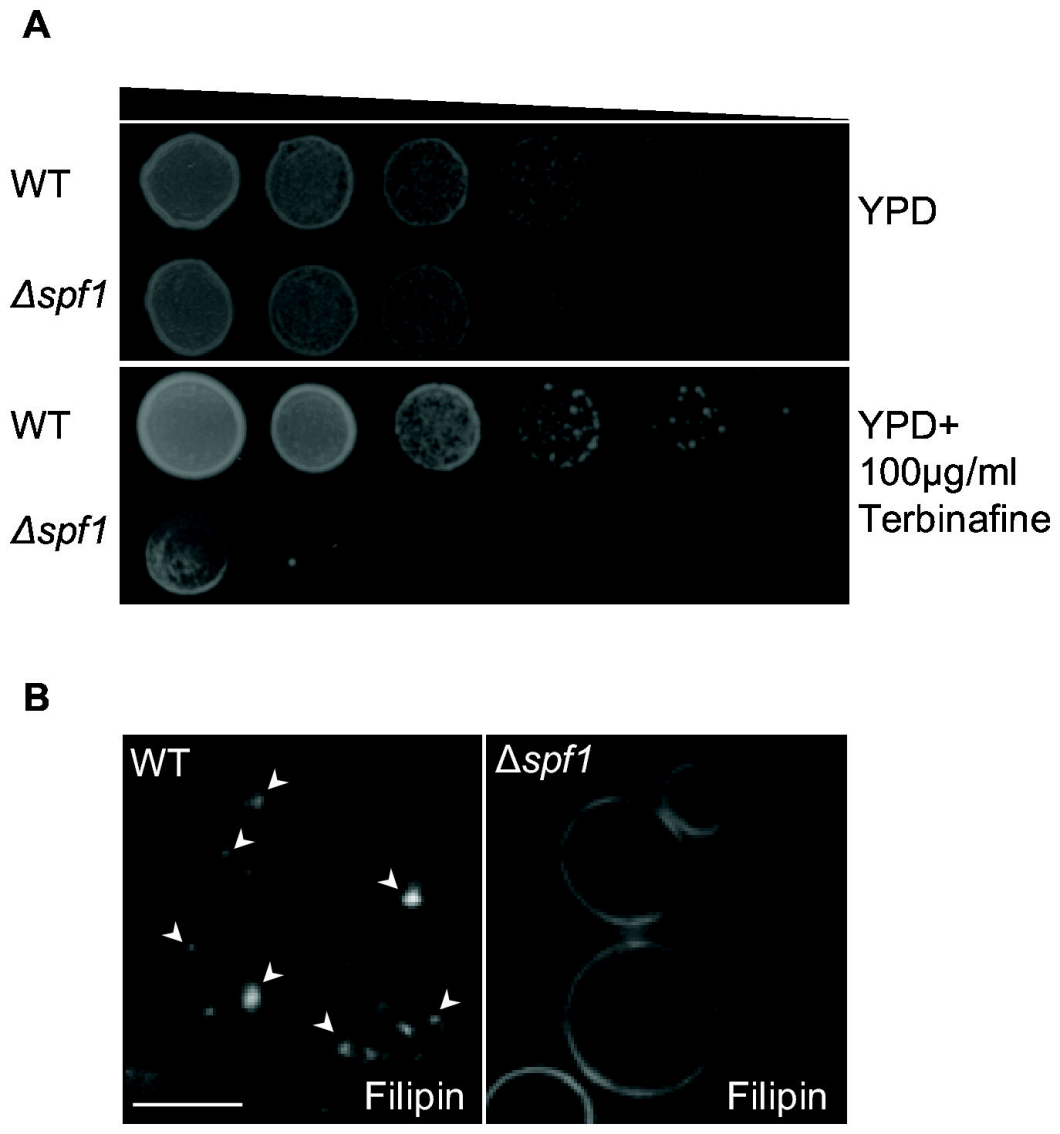
Δ*spf1* cells are more sensitive to the ergosterol biosynthesis inhibitor Terbinafine and display change in ergosterol distribution compared to WT. (**A**) WT and Δ*spf1* cells were plated in serial dilution on YPD plates without and with Terbinafine, an ergosterol biosynthesis inhibitor. Loss of SPF1 causes growth inhibition in the presence of this inhibitor. (**B**) WT and Δ*spf1* cells were stained for sterol distribution by fillipin staining. While most of the Δ*spf1* cells exhibit homogeneous sterol distribution, the WT shows a non-homogenous sterol staining pattern. Scale bar: 5µm.

Another lipid biosynthetic enzyme that is known to depend on Mn^2+^ for its function is the cytosolic farnesyl pyrophosphate (FPP) synthetase (Fpp1) in the sterol biosynthesis pathway [63,64]. Since this enzyme functions in the cytosol where higher levels of Mn^2+^ should be present in the ∆*spf1* background, we expected higher activity of this enzyme in ∆*spf1* cells. Overexpression of Fpp1 and hyperactivation of this pathway is known to cause a decrease in growth rate due to accumulation of squalene [65]. Indeed in the presence of the Erg1 inhibitor, Terbinafine [66], which slows down clearance of squalene, ∆*spf1* cells exhibit more severe growth defects than WT cells (Figure 3A). In addition we have found, both in this study and in previous ones, that WT and ∆*spf1* cells have very different subcellular distribution of sterols (Figure 3B) [27], which might also be a result of the increase in Fpp1 activity.

Our results hence show that known Mn^2+^-dependent enzymes in various lipid biosynthetic pathways are indeed affected in ∆*spf1* cells. We wished to examine if this property is general to a variety of other known Mn^2+^-dependent processes that can be followed in the cell.

### Exit of GPI-anchored proteins from the ER is affected in Δ*spf1* cells

**Figure 4:**
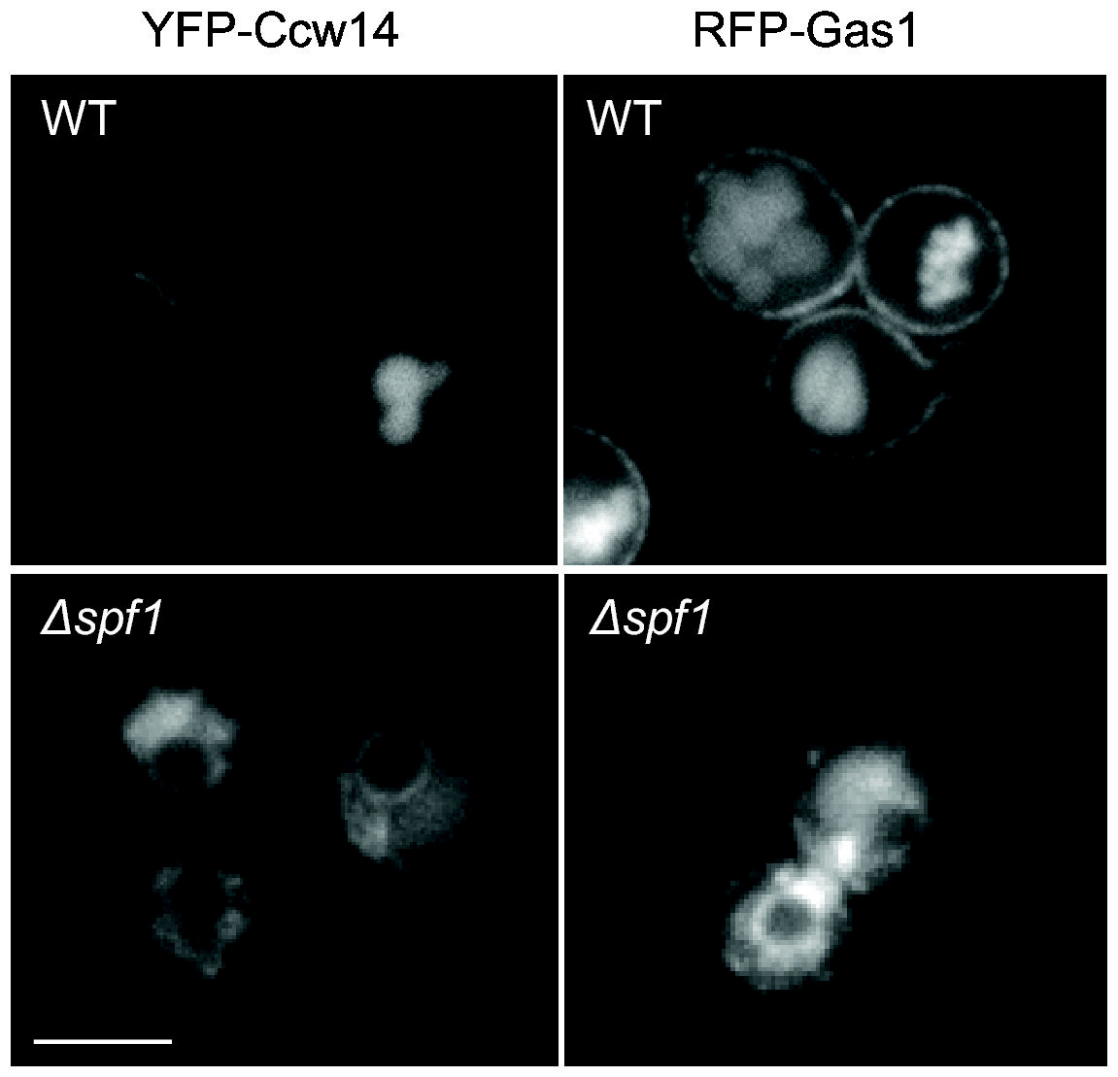
Spf1 affects the exit of GPI anchor proteins from the ER. WT and ∆*spf1* cells expressing tagged GPI anchored proteins (YFP-Ccw14 and RFP-Gas1) were imaged. While these proteins are normally localized to the cell periphery and the vacuole, a deletion of SPF1 enhances ER retention. Scale bar: 5µm.

Previous studies in mammalian cells [67] have shown that PGAP5 is an ethanolamine phosphate esterase that is involved in remodeling Glycosylphosphatidylinositol (GPI) anchors and that this step is required for ER exit of proteins that harbor such a lipid anchor. *S. cerevisiae* has two putative homologues of PGAP5, Cdc1 and Ted1. Since Cdc1 is Mn^2+^-dependent [68] it is likely that this holds true for Ted1 as well. Preventing any one of these remodeling reactions from occurring, should cause an accumulation of GPI-APs in the ER [69,70]. To assay if indeed GPI-AP exit is slowed down in *∆spf1* cells we imaged the fluorescently tagged GPI-APs, Gas1 and Ccw14 in this background. Both fusion proteins clearly exhibit an ER retention pattern in ∆*spf1* cells compared to the WT cells where they normally localize only to the cell periphery and the vacuole (Figure 4). 

### Protein Mannosylation is affected in ∆*spf1* cells

**Figure 5:**
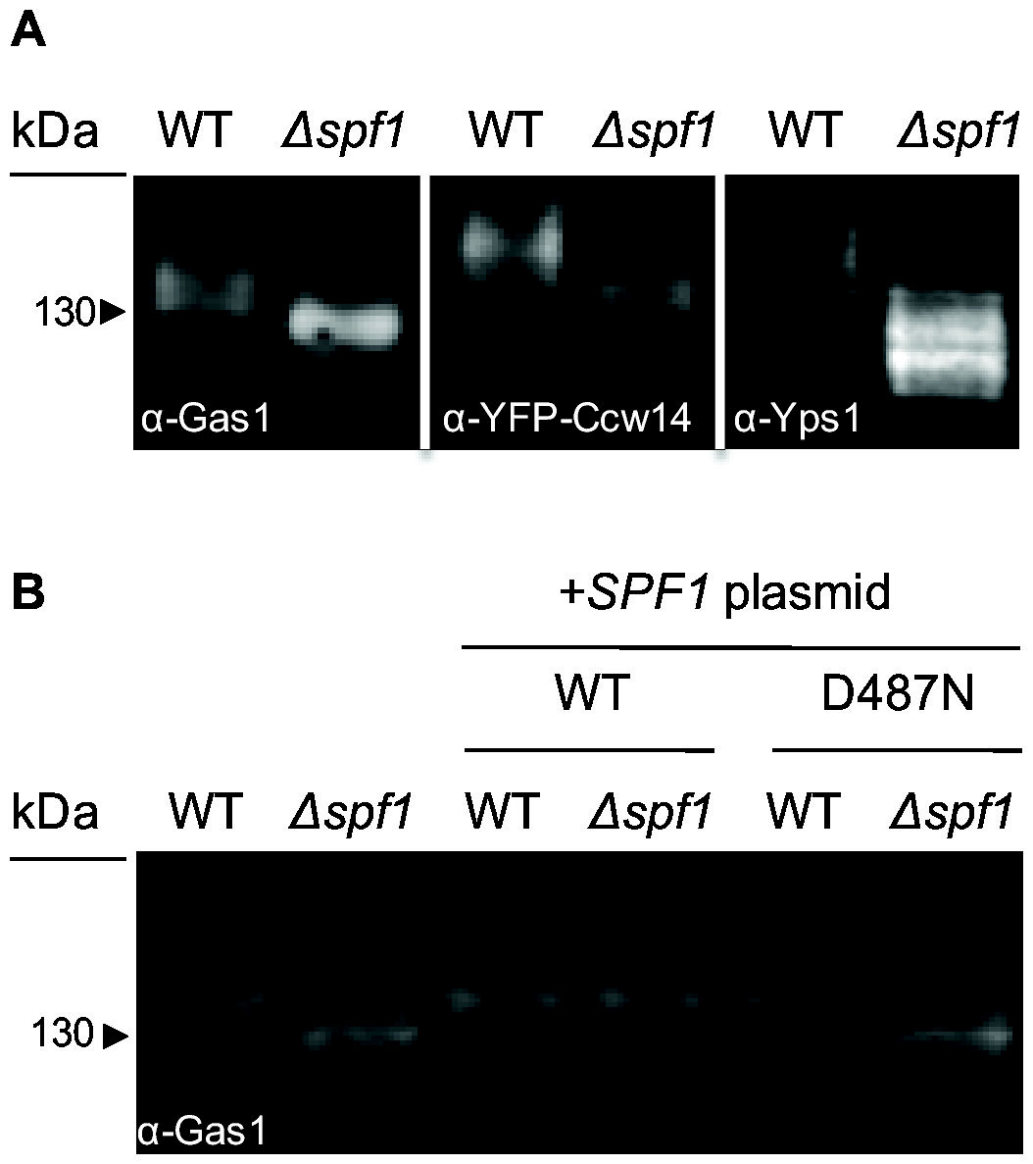
Spf1 affects protein mannosylation and its ATPase activity is required for its molecular function. (A) Western blot of three GPI anchored proteins that undergo known mannosylation (Gas1, Ccw14 and Yps1) reveals that this modification is reduced in Δ*spf1* compared to WT. (B) Western blot of Gas1 in WT and Δ*spf1* cells without any plasmid, with a plasmid containing full-length SPF1 gene, or with plasmid containing the full-length gene carrying a mutation rendering the protein ATPase-dead. Lack of rescue in the ATPase dead mutant confirms that the reason for the defect in Gas1 maturation is the absence of the functional ATPase activity of Spf1.

Two additional Mn^2+^-dependent enzymes are the alpha 1,2 mannosyltansferases, Ktr1 and Kre2 [71]. Changes in mannosylation should affect the molecular weight of secretory proteins. Indeed, western blot analysis of three secretory proteins (Gas1, Ccw14 and Yps1) revealed that their molecular weight is lower in ∆*spf1* cells compared to WT cells (Figure 5A). Alongside previous studies that have shown that glycosylation is affected in *∆spf1* strains [14,15] it is highly probable that both Ktr1 and Kre2 are hypo-functional in this background.

### The ATPase activity of Spf1 is required for its function

To ensure that the phenotypes observed in the deletion of the *SPF1* gene are indeed a result of loss of its ATPase activity we established a rescue assay by transforming ∆*spf1* cells with a plasmid containing either the full-length *SPF1* gene or the *SPF1* gene mutated in essential residues for the ATPase activity (D487N) [13,14,27]. In this mutant protein the aspartate residue (D) in the phosphorylation site, which is known to be strictly conserved in all P-type ATPases, was replaced by an asparagine residue (N) Assaying a phenotype such as the glycosylation pattern of Gas1 in these strains demonstrated that it is indeed the ATPase activity and therefore the transport function of this protein, that is essential for its activity in the cells (Figure 5B). 

### ATP13A1 is the functional homologue of Spf1

The importance of Spf1 is underscored by the presence of conserved homologues in all higher eukaryotes: worms, insects, fish, mice and humans. Previous studies that have characterized the family of mammalian P5 ATPases indentified five genes, ATP13A1-ATP13A5, that belong to this family of ATPases due to their high degree of homology to the yeast P5 ATPases, Spf1 and Ypk9. Amino acid sequence comparisons between these five mammalian genes and the two yeast genes suggested that ATP13A1 is orthologous to the yeast *SPF1* gene and that ATP13A2-5 are orthologous to the yeast *YPK9* gene [72]. However, until now the functional homology has not been experimentally validated and in fact nearly no information exists regarding ATP13A1 except for its mRNA expression pattern that peaks at the height of neurogenesis [73]. 

**Figure 6:**
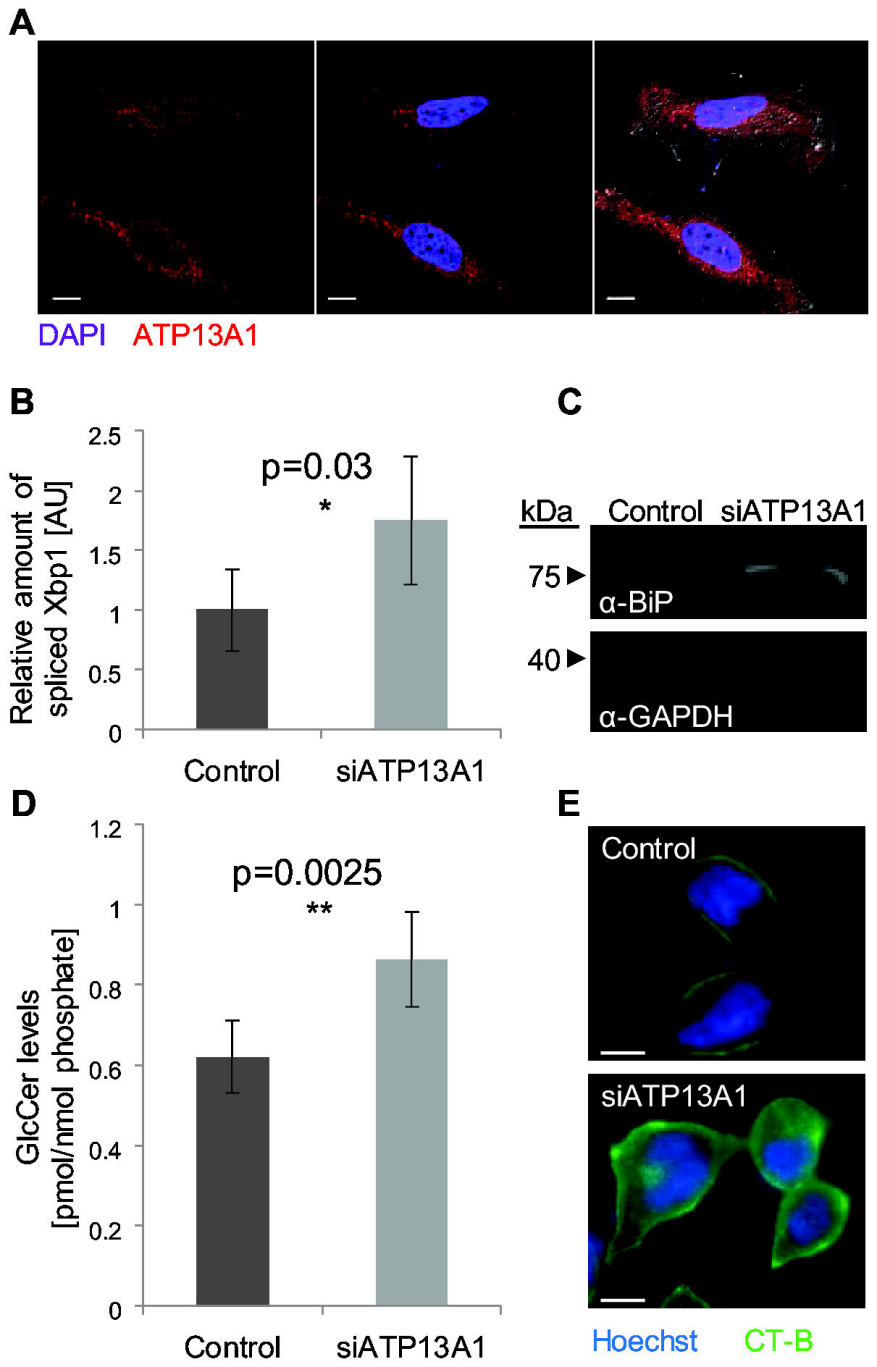
ATP13A1 is the functional homologue of Spf1. (**A**) Immunostaining of HeLa cells with α-ATP13A1 and DAPI (nucleus staining) indicates perinuclear localization for ATP13A1. Scale bar: 10µm. (**B**) Amount of spliced XBP1 (a result of ER stress) was assayed by qPCR analysis. Silencing of ATP13A1 in HeLa cells causes an increase in the spliced form. (**C**) Western blot of BiP protein reveals that its amount is increased upon silencing of ATP13A1 in HeLa cells. Blot was also probed with an antibody against GAPDH as a loading control. (**D**) Mass Spectrometry analysis reveals a specific increase in GlcCer levels in cells silenced for ATP13A1 suggesting enhanced activity of GlcCer synthase. (**E**) Fluorescent cholera toxin subunit B (CT-B) was used to stain the plasma membrane ganglioside GM1. Cells treated with an siRNA against ATP13A1 display accumulation of GM1. Scale bar: 10µm.

In order to characterize ATP13A1 we first wanted to verify that it is distributed in the cell in a similar manner to Spf1. Indeed, immunohistochemistry of HeLa cells reveals a reticular pattern surrounding the nucleus of the cell as would be expected from an ER resident protein (Figure 6A).

In yeast, it has been shown that the absence of Spf1 causes induction of the unfolded protein response (UPR) [12]. To measure whether loss of ATP13A1 results in UPR induction, we silenced ATP13A1 by a pool of siRNAs and measured XBP1 splicing and levels of the major ER chaperone BiP (which are induced upon ER stress and serve as markers for its occurrence) [74,75]. Indeed HeLa cells silenced for ATP13A1 exhibit high levels of the spliced form of XBP1 and higher protein levels of BiP compared to the control of non targeting (scrambled) siRNA pool (Figure 6B,C) demonstrating a conservation of ATP13A1’s importance for ER functions. 

Finally, glucosylceramide (GlcCer) synthase [76], a Golgi-associated protein whose Mn^2+^-dependent active site faces the cytosol, displays increased activity in cells depleted for ATP13A1 in a manner consistent to the function of Spf1 in yeast. Specifically, direct measurement of GlcCer levels by mass spectrometry demonstrated their accumulation in the ATP13A1 silenced cells (Figure 6D) and labeling of downstream metabolites of GlcCer such as the ganglioside GM1, which can be detected using cholera toxin (CT) [77] revealed increased GM1 in these cells (Figure 6E). 

Taken together, these observations strengthen the determination that ATP13A1 is the human functional orthologue of Spf1.

## Discussion

*SPF1* was originally discovered following a screen for salt-mediated killer toxin (SMKT) resistant mutants and was defined as a putative P-type ATPase that belongs to the poorly characterized P5 subfamily of these transporters [13]. Since then this gene was detected in a large number of additional screens demonstrating its importance for a variety of cellular functions (Table S1) however, the molecular function of Spf1 in the ER remained unknown. Therefore, the underlying reason for the various phenotypes of *SPF1* mutants, and whether they are direct or secondary, was not understood.

Due to the fact that Spf1 belongs to the ATPase transporter family, that the ER Mn^2+^ transporter has not yet been identified, as well as a recent study that claims a role for the second yeast P5-ATPase, Ypk9, in Mn^2+^ homeostasis [37], it is alluring to speculate that Spf1 is the ER Mn^2+^ transporter. Unfortunately, despite the dramatic increase in chemical and genetically encoded reporters for many ions [78], there are still no reporters for Mn^2+^. As so until now the functional studies on Spf1 utilized an *in vitro* reconstitution strategy that has not yielded much insight on its substrate specificity [33,36]. Interestingly, in these previous studies the ATPase activity of Spf1 was very specifically affected by addition of Mn^2+^ and no other bivalent ion. However, since Mn^2+^ addition reduced transporter activity rather than induced it this was not considered the effect of a direct substrate. To overcome the measurement hurdles we decided to take a different approach by performing specific *in vivo* experiments in which we measured the direct (reduction of Mn^2+^ levels in microsomes) and indirect (changes in localization of the Mn^2+^ sensors and reduction in activity of Mn^2+^ dependent enzymes) effects of deleting *SPF1*. Although *in vivo* experiments can never prove that an ion is a direct transportee of a transporter, our studied enable us to conclude that Spf1 has an important role in Mn^2+^ homeostasis in the cell. Our analysis of Mn^2+^ requiring enzymes depended on previous biochemical studies regarding activities of the various enzymes and their co-factor requirements [58,63,64,68,71,76,79,80]. To this end, we may have missed additional Mn^2+^-dependent enzymes in the ER or cytosol that have not yet been characterized. However, even by merely assaying these enzymes we can already see that many of the phenotypes displayed by loss of *SPF1* can be explained through modulation of Mn^2+^-dependent enzyme function.

Surprisingly, we could also see an affect of Spf1 loss on Golgi localized enzymes: Aur1 and Kre2/Ktr1. Given that a Mn^2+^ transporter, Pmr1, has been suggested for the Golgi [81], this influence on Golgi enzymes is surprising. We see two possible explanations for these observations:

1 Since the lumen of the Golgi apparatus is constantly being created from ER derived vesicles [82] major changes in the composition of the ER lumen should influence Golgi composition. Lack of ER luminal Mn^2+^ should therefore result in dilution of this ion in the Golgi apparatus as well.
2 The Golgi is divided into cis, medial and trans parts. If Pmr1 resides in the trans Golgi, then early Golgi compartments (which may be the specific Golgi localization of Kre2/Ktr1 and Aur1) might not be affected by its function.

Many of the Mn^2+^-dependent enzymes are essential and thus the wide spread effect of losing *SPF1* is clear. However, it is interesting to note that *SPF1* itself is not an essential gene for the survival of a cell. The reason for this may lie in the fact that most of these Mn^2+^-dependent enzymes can also utilize other, similar, ions as co-factors, but at a much lower efficiency; or that in the absence of Spf1 retrograde traffic to the ER enables a small flow of Mn^2+^ from later secretory pathway organelles that have their own transporters. 

Close homologues of Spf1 exist in several metazoans. A previous study demonstrated that Spf1 is functionally conserved between yeast and *Drosophila melanogaster* [33]. In view of the central role that Spf1 has in yeast and flies we thought that it is also important to identify its mammalian functional homologue. Although ATP13A1 was described as the sequence homologue of *SPF1* already ten years ago [72], this homology was not yet examined at the level of protein activity. Now that we proposed a specific molecular function for Spf1 in the yeast ER, it becomes possible to examine if this activity is also shared by ATP13A1 in mammalian cells. Indeed, our experiments on localization, UPR induction and affect on the function of a Mn^2+^-dependent enzyme, all support a functional conservation for these two enzymes. Although never studied, it is clear that ATP13A1 is an important factor in the nervous system due to the fact that its expression peaks at the height of neurogenesis and is strongest in several brain regions of adult mouse [73]. Since changes in Mn^2+^ levels have a wide and established role in neurodegenerative diseases [83] our studies open the way for important studies on this proteins’ role in health and diseases of the nervous system. 

More generally, the ER is an essential organelle that is important for the function of all eukaryotic cells. Despite this, the molecular function of more than a third of its proteins remains unknown [11]. Since loss of *SPF1* induced severe ER stress, it was obvious that its molecular function must be characterized in order to better understand ER physiology in general. Our studies, supporting a role for Spf1 in Mn^2+^ homeostasis, shed light on its widespread effects and the various phenotypes that ∆*spf1* cells exhibit. In a more global view, our research demonstrates how studying a simple eukaryote model organism as the yeast cell, can contribute to better understanding of basic cellular processes and the function of homologous genes in higher eukaryotes.

## Supporting Information

Table S1 **Description of screens in which ∆*spf1* was detected as a hit (by chronological order).**
(DOC)

Table S2 ***Saccharomyces cerevisiae* strains used in this study.**
(DOC)

Table S3 **Plasmids used in this study.**
(DOC)

Table S4 **Primers used in this study.**
(DOC)
