# Supplementary material for: The Yeast P5 Type ATPase, Spf1, Regulates Manganese Transport into the Endoplasmic Reticulum
Source: PLoS One. 2013 Dec 31;8(12):e85519. doi: 10.1371/journal.pone.0085519 (PMC3877380; doi:10.1371/journal.pone.0085519)
Supplement: Table S1 — Description of screens in which ∆spf1 was detected as a hit (by chronological order). (DOC) [file pone.0085519.s001.doc]

**Table S1. Description of screens in which ∆*spf1* was detected as a hit (by chronological order).**

| Screen description | Reference |
| --- | --- |
| Resistance for salt-mediated killer toxin (SMKT) | [13] |
| Affects on degradation of the HMG-CoA reductase, Hmg2 | [16] |
| Defects in protein insertion orientation (PIO) | [17] |
| A genetic interaction map in which a *∆spf1* strain clustered together with strains mutated for components of the posttranslational translocation machinery | [18] |
| Sensitivity to UPR-inducing agents | [19,20] |
| Sensitivity to Oleate | [21] |
| Tolerance to Air-drying | [22] |
| Requirement for protein folding in the ER | [12] |
| Sensitivity to arsenic toxicity | [23] |
| Requirement for maximal tolerance to acetic acid | [24] |
| Requirement for resistance to the antimalarial drug quinine | [25] |
| Requirement for zinc-limited growth | [26] |
| Affects on targeting of tail anchor proteins | [27] |
