# Supplementary material for: The Yeast P5 Type ATPase, Spf1, Regulates Manganese Transport into the Endoplasmic Reticulum
Source: PLoS One. 2013 Dec 31;8(12):e85519. doi: 10.1371/journal.pone.0085519 (PMC3877380; doi:10.1371/journal.pone.0085519)
Supplement: Table S2 — Saccharomyces cerevisiae strains used in this study. (DOC) [file pone.0085519.s002.doc]

**Table S2. *Saccharomyces cerevisiae* strains used in this study.**

| Strain name | Genotype | Background | Source (Reference) |
| --- | --- | --- | --- |
| WT | MATa *his3*∆1 *leu2*∆0 *met15*∆0 *ura3*∆0∷KanR | BY4741 | ATCC |
| ∆*spf1* | MATa *his3*∆ *leu2*∆0 *met15*∆0 *ura3*∆0 ∆*spf1*::KanR | BY4741 | [43] |
| OE-Spf1 | MATa *his3*∆ *leu2*∆0 *met15*∆0 *ura3*∆0 NatR::Tef2pr-3HA-Spf1 | BY4741 | This study |
| GFP-Smf1 | MATa *his3*∆1 *leu2*∆0 *met15*∆0 *ura3*∆0∷KanR NatR::Tef2pr-GFP-Smf1 | WT | This study |
| ∆*spf1* GFP-Smf1 | MATa *his3*∆ *leu2*∆0 *met15*∆0 *ura3*∆0 ∆*spf1*::KanR NatR::Tef2pr-GFP-Smf1 | ∆*spf1* | This study |
| GFP-Smf2 | MATa *his3*∆1 *leu2*∆0 *met15*∆0 *ura3*∆0∷KanR NatR::Tef2pr-GFP-Smf2 | WT | This study |
| ∆*spf1* GFP-Smf2 | MATa *his3*∆ *leu2*∆0 *met15*∆0 *ura3*∆0 ∆*spf1*::KanR NatR::Tef2pr-GFP-Smf2 | ∆*spf1* | This study |
