# Supplementary material for: The Yeast P5 Type ATPase, Spf1, Regulates Manganese Transport into the Endoplasmic Reticulum
Source: PLoS One. 2013 Dec 31;8(12):e85519. doi: 10.1371/journal.pone.0085519 (PMC3877380; doi:10.1371/journal.pone.0085519)
Supplement: Table S3 — Plasmids used in this study. (DOC) [file pone.0085519.s003.doc]

**Table S3. Plasmids used in this study.**

| Plasmid name | Description | Yeast marker | Source (Reference) |
| --- | --- | --- | --- |
| pYM-N21-natNT2-TEF-yeGFP | Used for N-terminally GFP-tagging of Smf1/2 and to replace the native promoter with the TEF promoter. | NatR | [44] |
| pRS416 RFP-Gas1 | Used for detecting the localization of yeast GPI-anchored proteins | *URA3* | Kindly provided by Howard Riezman |
| pRS416 YFP-Ccw14 | Used for detecting the localization of yeast GPI-anchored proteins | *URA3* | [45] |
| pSM2 SPF1-HA | Used for expressing Spf1 in rescue assays | *LEU2* | Kindly provided by Davis Ng |
| pSM2 SPF1-HA D487N | Used for expressing Spf1 ATPase mutant in rescue assays | *LEU2* | [27] |
