# Supplementary material for: The Yeast P5 Type ATPase, Spf1, Regulates Manganese Transport into the Endoplasmic Reticulum
Source: PLoS One. 2013 Dec 31;8(12):e85519. doi: 10.1371/journal.pone.0085519 (PMC3877380; doi:10.1371/journal.pone.0085519)
Supplement: Table S4 — Primers used in this study. (DOC) [file pone.0085519.s004.doc]

**Table S4. Primers used in this study.**

| Primer name | Sequence | Purpose |
| --- | --- | --- |
| Smf1-N'GFP tag-F | ATATATCAGCAGAAAAACTAACTTTCTCAATTAGGTCAAAATGcgtacgctgcaggtcgac | Forward primer for N-terminal tagging of Smf1, based on pYM plasmids |
| Smf1-N'GFP tag-R | CATCCACAGCAACTGCAGCATGAGAAGGACCAACGTTCACcatcgatgaattctctgtcg | Reverse primer for N-terminal tagging of Smf1, based on pYM plasmids |
| Smf2-N'GFP tag-F | GCCATTTAACCACTAATTTTCCGTATAGCGTTTATGTTTGATGcgtacgctgcaggtcgac | Forward primer for N-terminal tagging of Smf2, based on pYM plasmids |
| Smf2-N'GFP tag-R | ATTCATCGCTCCATTGAATAGGTTCATATTCTTGGGACGTcatcgatgaattctctgtcg | Reverse primer for N-terminal tagging of Smf2, based on pYM plasmids |
| Smf1-N'TagCHK-F | AGAGCCTTGTTAACGCGCTC | Forward primer for chking N-terminal tagging of Smf1 |
| Smf2-N'TagCHK-F | ACCTCTTCCTTACAAGAAGC | Forward primer for chking N-terminal tagging of Smf2 |
| S1-chk-R | GTCGACCTGCAGCGTACG | Reverse primer for chking N-terminal tagging made using pYM plasmids |
| spf1-wtchk-F | AGAGCACGCTGTTGCGCCAT | Forward primer for chking existence of spf1 wt copy |
| spf1-wtchk-R | AATTGCGCTCTCCTCGGCGG | Reverse primer for chking existence of spf1 wt copy |
| act1-chk-F | TGTCACCAACTGGGACGATA | Forward primer for chking existence of act1 wt copy, which used as reference |
| act1-chk-R | GGCTTGGATGGAAACGTAGA | Reverse primer for chking existence of act1 wt copy, which used as reference |
| atp13a1-qpcr-F | AAGCCGGTTGACAGCGGGTG | Forward primer for QPCR of human atp13a1 |
| atp13a1-qpcr-R | GCAGCTTGCCCTGGGATGTGT | Reverse primer for QPCR of human atp13a1 |
| XBP1sp1-F | TGCTGAGTCCGCAGCAGGTG | Forward primer to differentiate between two transcript of human xbp1 |
| XBP1sp1-R | GCTGGCAGGCTCTGGGGAAG | Reverse primer to differentiate between two transcript of human xbp1 |
